# Drought stress stimulates endocytosis and modifies membrane lipid order of rhizodermal cells of *Medicago truncatula* in a genotype-dependent manner

**DOI:** 10.1186/s12870-019-1814-y

**Published:** 2019-05-28

**Authors:** Mégane Couchoud, Christophe Der, Sylvie Girodet, Vanessa Vernoud, Marion Prudent, Nathalie Leborgne-Castel

**Affiliations:** 1 Agroécologie, AgroSup Dijon, INRA, University of Bourgogne, University of Bourgogne Franche-Comté, F-21000 Dijon, France; 2 Agroécologie, AgroSup Dijon, CNRS, INRA, University of Bourgogne, University of Bourgogne Franche-Comté, F-21000 Dijon, France

**Keywords:** *Medicago truncatula*, Root architecture, PEG, Endocytosis, Order degree of the membrane, Fluorescent dyes

## Abstract

**Background:**

Drought stress negatively affects plant growth and productivity. Plants sense soil drought at the root level but the underlying mechanisms remain unclear. At the cell level, we aim to reveal the short-term root perception of drought stress through membrane dynamics.

**Results:**

In our study, 15 *Medicago truncatula* accessions were exposed to a polyethylene glycol (PEG)-induced drought stress, leading to contrasted ecophysiological responses, in particular related to root architecture plasticity. In the reference accession Jemalong A17, identified as drought susceptible, we analyzed lateral roots by imaging of membrane-localized fluorescent probes using confocal microscopy. We found that PEG stimulated endocytosis especially in cells belonging to the growth differentiation zone (GDZ). The mapping of membrane lipid order in cells along the root apex showed that membranes of root cap cells were more ordered than those of more differentiated cells. Moreover, PEG triggered a significant increase in membrane lipid order of rhizodermal cells from the GDZ. We initiated the membrane analysis in the drought resistant accession HM298, which did not reveal such membrane modifications in response to PEG.

**Conclusions:**

Our data demonstrated that the plasma membranes of root cells from a susceptible genotype perceived drought stress by modulating their physical state both via a stimulation of endocytosis and a modification of the degree of lipid order, which could be proposed as mechanisms required for signal transduction.

**Electronic supplementary material:**

The online version of this article (10.1186/s12870-019-1814-y) contains supplementary material, which is available to authorized users.

## Background

Climate change is expected to cause more frequent and more intense episodes of drought [1]. Unfortunately, drought-induced injury is a major constraint in agriculture that limits geographic distribution and productivity of crop plants [2]. To cope with drought, plants have developed several strategies under natural selection, including numerous changes at the morphological, physiological, and biochemical levels in all plant organs (for review [3]).

Plant roots are essential organs that provide the plant with water and nutrients. They also represent the primary site for sensing drought and initiating signaling cascades to the whole plant for adequate responses. However, due to limitations on root accessibility, the influence of drought on this organ has been much less studied than aerial plant parts. Roots are organized in a fixed radial pattern of different cell types that persist throughout the root axis. The outermost cell layer is the rhizodermis that functions as a selective barrier between the root and its environment. Below is the cortex that consists of a single cell layer as in *Arabidopsis* or of several [4–6] cell layers as in *Medicago truncatula* [4]. The deepest cells are the vascular bundle cells of the stele that are surrounded by single layers of pericycle and endodermis cells. Roots also display a developmental gradient along their longitudinal proximo-distal axes, with young cells being close to the root tip and the older mature cells at the root base [5]. Therefore, depending on their age, location and/or identity, root cells have diverse forms and shapes associated with specific functions for their development and stress responses [6]. During drought, one of the plant adaptations for survival is the adjustment of the root system architecture to maximize water absorption [7], but little is known about how the root perceives soil drought at an early stage to promptly respond to water stress.

The plasma membrane (PM) is the primary site of perception for responding to external abiotic stimuli [8]. Adverse environments could negatively impact the PM and this feature has been widely used to evaluate the extent of cell damage [9]. In particular, intense drought stress causes disturbance of the cell membrane leading to a loss of membrane integrity [10]. However, osmotic stress triggered by moderate water stress may modify the physical properties of membrane lipids that can be perceived by cells via sensory proteins anchored on the PM, such as receptor kinases or mechanosensitive ion channels [11]. Even though direct drought sensors have not been yet identified, environmental signals are transferred to networks of transduction pathways, with the resulting regulation of gene expression.

The barrier function of the PM and PM plasticity are thus influenced by the physical state of lipid bilayers that may make the membrane (or the cell) resistant or susceptible to environmental changes [12–14]. Indeed, PM plasticity, which corresponds to dynamics either by lateral compartmentalization or intracellular trafficking of membrane molecules, modulates the perception and transduction of environmental cues [15]. The PM contains microdomains of specific lipid composition that influence the PM protein dynamics [16, 17]. PM protein homeostasis also depends on recycling and/or degradation, two processes that are initiated by endocytosis [18].

The mechanisms underlying early cellular responses to drought impact are little studied. Their elucidation would help us to better exploit legumes, which represent a sustainable and valuable food source for humans and animals [19]. Recent studies on the impact of drought stress on the alfalfa (*Medicago sativa* L.) proteome showed a stress-induced adaptation of the plant notably by increasing the quantity of membrane proteins such as those involved in membrane trafficking or membrane modification [20–22].

One standard way to induce drought is the incorporation of polyethylene glycol (PEG) of higher molecular weight (4000 to 8000) in root medium. This non-absorbable and non-metabolized osmotic agent induces moderate water stress by modifying the osmotic potential in a controlled manner [23]. By making less water available to the plant roots, as in a dry soil, PEG solutions are well-suited to simulate water deficit and plants are able to increase their water absorption area by stimulating lateral root (LR) development [24, 25].

In the present study, we have analyzed the impact of PEG treatment on the model legume *Medicago truncatula*. Fifteen *Medicago truncatula* accessions were phenotyped for their response to PEG treatment in a growth pouch experiment, allowing the study of overall plant growth and root architecture. On the reference accession Jemalong A17, we detected a very early (short-term) perception of PEG-induced drought stress at LR membranes of seedlings. For that purpose, we used the FM4–64 and di-4-ANEPPDHQ fluorescent probes, to monitor endocytosis and lipid order, respectively. We showed that PEG stimulated a rapid endocytosis in rhizodermal cells within 10 min and over a more prolonged period in cortical cells. We also observed a longitudinal gradient of stimulated endocytosis from root caps to elongated cells where the more the cell is mature, the more it responds. Similarly, membranes of rhizodermal cells, which presented a lower lipid order that cortical cells, were less rigid in differentiated/mature zones. In contrast, in this differentiated zone (/mature zone) the rhizodermal cells responded to PEG by increasing lipid order of their membranes. Finally, we extended our analysis to a second accession, which we showed to be PEG-resistant.

## Results

### PEG applied to a core-collection of *Medicago truncatula* revealed accession-dependent responses

**Fig. 1:**
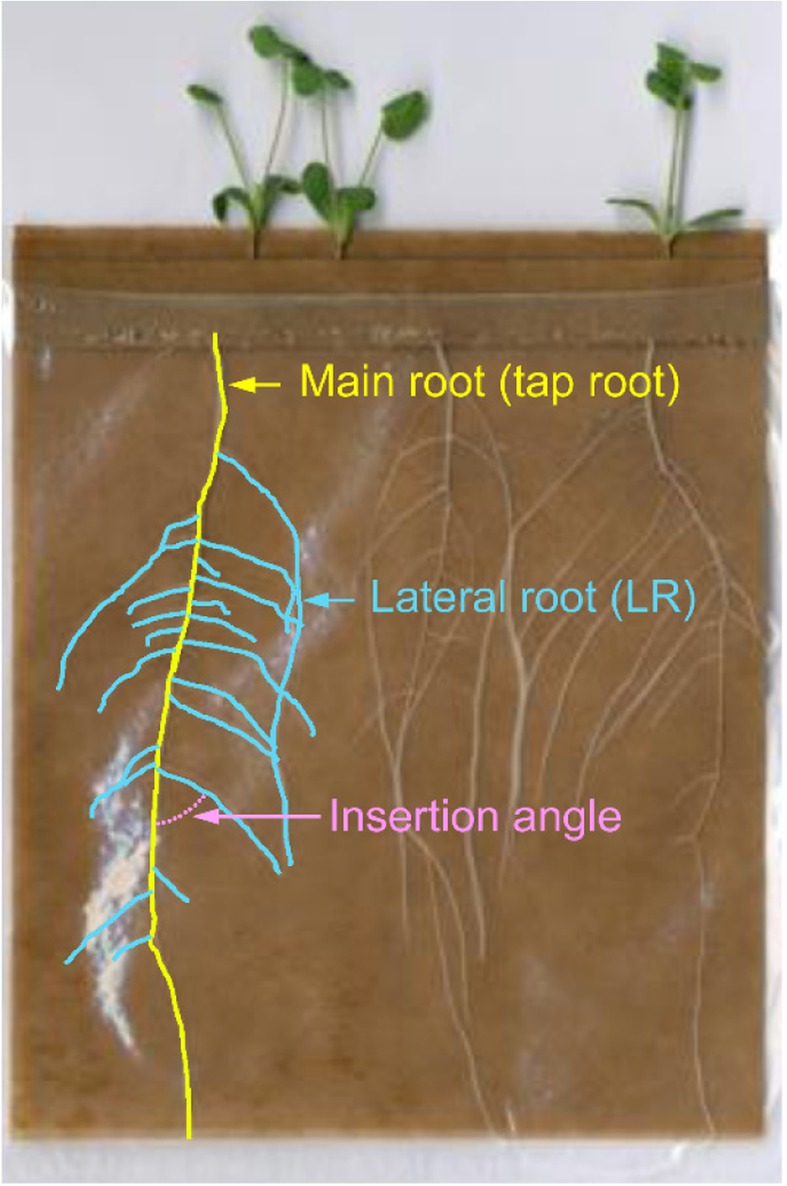
Growth of *Medicago truncatula* in pouches. For each accession, three seedlings per pouch (five pouches per accession and per treatment) were grown either under untreated or PEG-treated conditions. After root system scanning, the main root and lateral roots were identified and architectural traits were measured (see Table 1 and Additional file 1: Table S1)

A panel of 15 *Medicago* accessions (Table 1 and Additional file 1: Table S1) was evaluated for its response to PEG-induced drought stress. This panel included 12 inbred lines out of the 16 that constitutes a nested core collection representative of *Medicago truncatula* genetic diversity (CC16, [26]), the Jemalong A17 reference accession used for the genome sequencing effort (hereafter named A17), and two accessions (HM026 and HM298) that were previously tested in an in vitro leaf dehydration assay using PEG and that displayed a contrasted response [27]. Seedlings were grown in plastic pouches and treated for 5 days with 15% PEG 8000 (Fig. 1). With this system, root growth could be easily monitored through the transparent plastic pouch, allowing us to analyze specific root architecture traits such as total projected root length, specific root length, LR number and LR insertion angle.

**Table 1:**
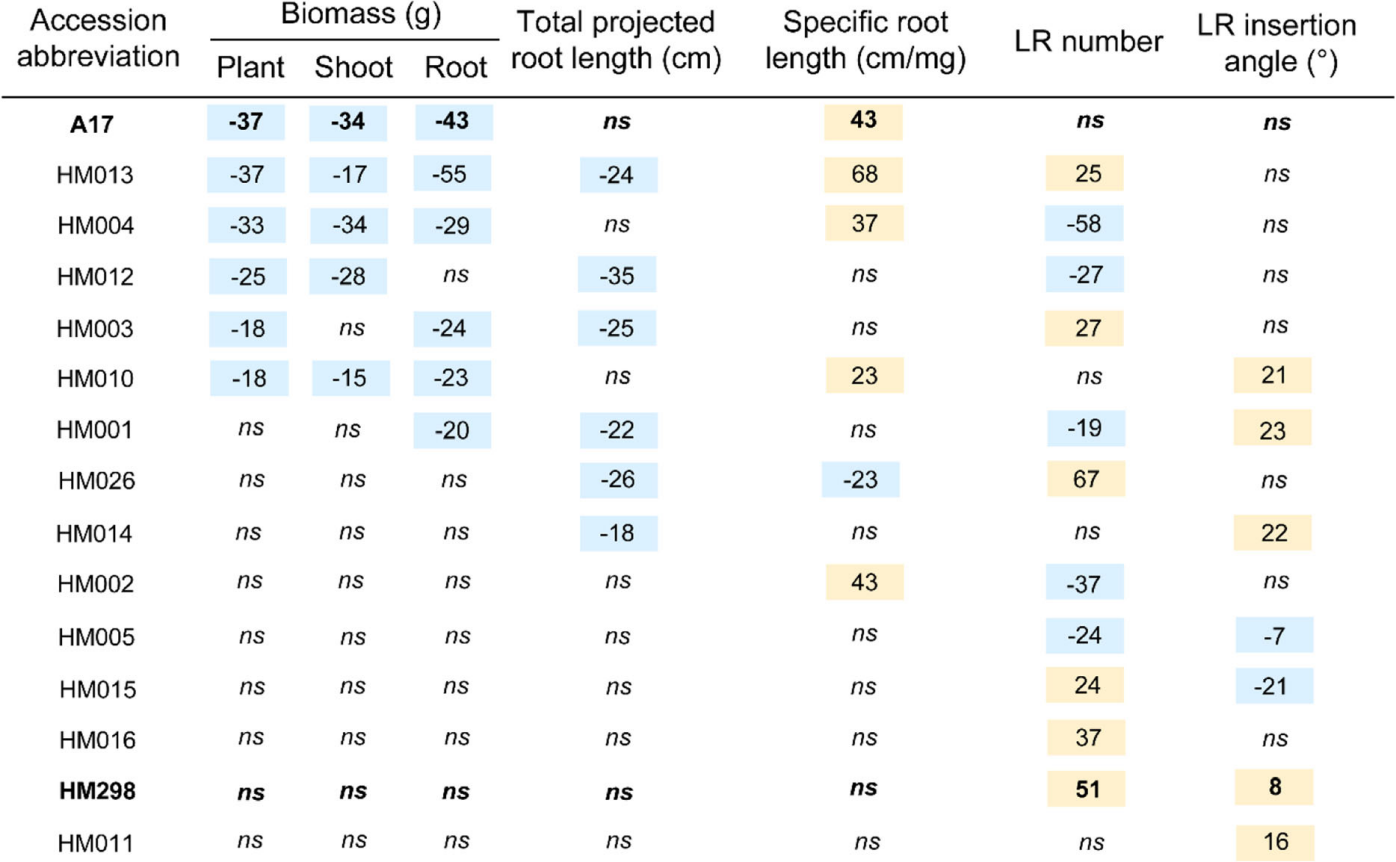
Growth of *Medicago truncatula* accessions after 12 days in pouches: impact of PEG treatment on biomasses and root architectural traits. For each accession, the impact of 8-days PEG treatment on each trait was expressed as a percentage relative to the untreated condition. Values are given in Additional file 1: Table S1. Negative and positive values indicate that PEG decreased or increased the value of the trait, respectively. *ns* means that impact of PEG treatment was not significant at the 0.05 probability level. The two selected and studied genotypes in the following experiments are highlighted in bold.

Each of the 15 accessions displayed specific responses to drought (Table 1). Among the 15 accessions, only six significantly lost plant biomass (ranging from − 18% in HM003 and HM010 to − 37% in A17 and HM013) following PEG treatment, and were thus considered as PEG-susceptible. This biomass loss was the result of either combined decreased shoot and root biomasses (A17, HM004, HM010, HM013), or biomass decrease localized to the shoot (HM012) or to the root system (HM003 and HM001). Concomitantly with these biomass losses, architectural traits were impacted by PEG application although no common trend was shared between the PEG-susceptible accessions. While the lateral root number decreased in response to PEG treatment for accessions HM004, HM012 and HM001, it increased for accessions HM013 and HM003 and this trait was not affected in A17 and HM010. Total root length was negatively affected by PEG application in four (HM013, HM012, HM003, HM001) out of the seven PEG-susceptible accessions while the average insertion angle of lateral roots was increased in two of them (HM010, HM001). In four PEG-susceptible accessions (A17, HM013, HM004, HM010), specific root length (SRL) significantly increased in response to PEG while it was not affected in the other three accessions.

On the other hand, in eight accessions, PEG application induced changes in morphological traits of the root system without impacting biomass acumulation. These accessions were thus considered as PEG-resistant, and included the HM298 and HM026 accessions previously ranked among the most resistant accessions in a PEG-induced leaf dehydration assay [27]. Lateral root number was increased by PEG in four accessions (HM026, HM298, HM015 and HM016), concomitantly (HM026) or not (HM298, HM015 and HM016) with a decrease in the total root length, and concomitantly (HM014, HM005, HM015, HM298, HM011) or not (HM002 and HM016) with changes of the LR insertion angle.

### PEG-stimulated endocytosis depends on root cell types

**Fig. 2:**
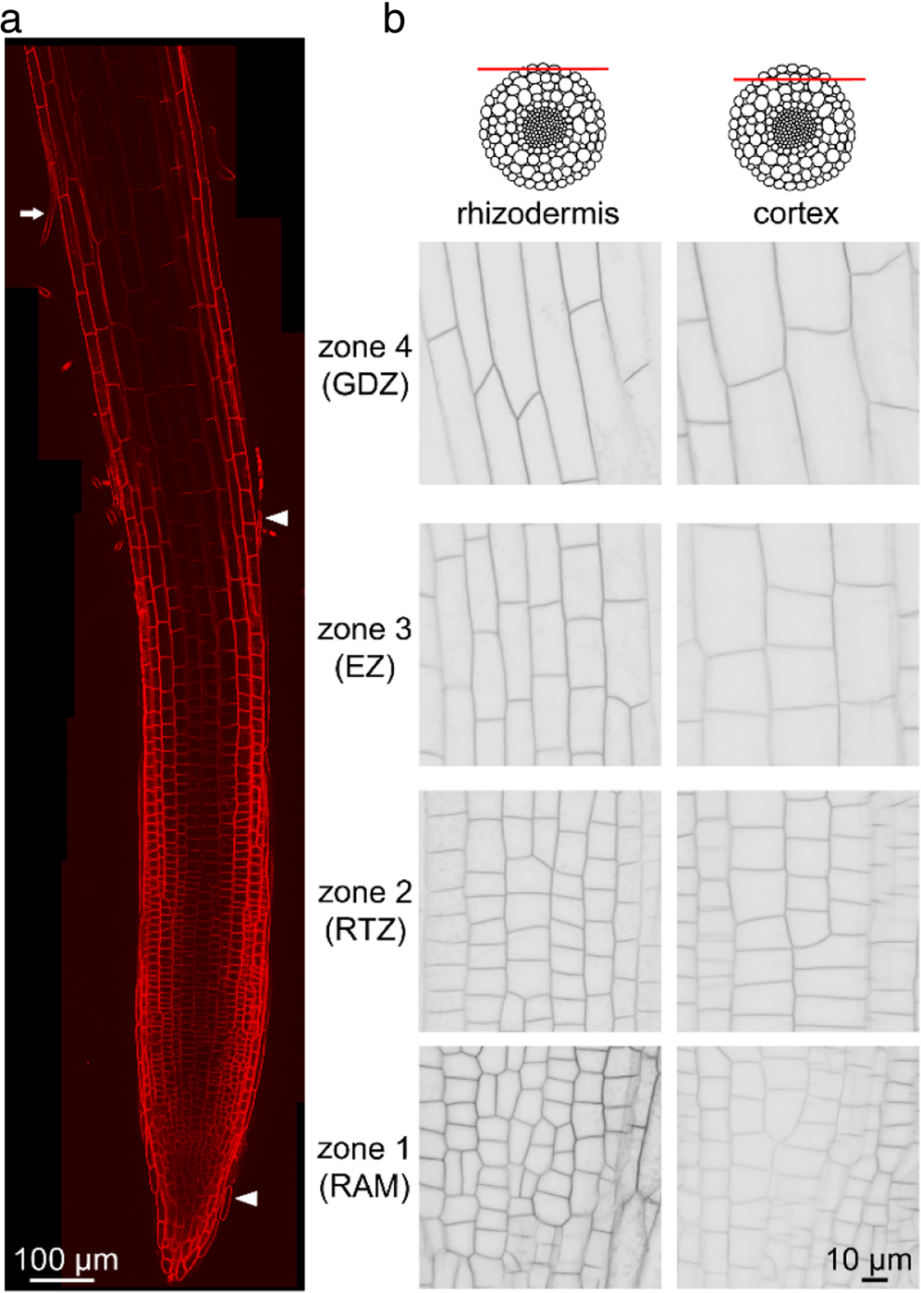
FM4–64 membrane labeling allowed the distinction of cells from LR apex of accession A17. **a** Confocal image of FM4–64 labelled LR. The four LR functional zones are distinghed as zone 1: root cap and meristematic zones (RAM), zone 2: root transition zone (RTZ), zone 3: elongation zone (EZ), and zone 4: growth differentiation zone (GDZ). Arrowheads: detached root cap cells; arrow: root hair in GDZ. **b** Detail of rhizodermal and cortical cells in the four functional zones labeled with FM4–64. Grayscale tone of confocal images of the root layers

We aimed first at analyzing endocytosis by using the FM4–64 fluorescent dye, which embeds into the membrane. Collected lateral root apices (~ 1.5 to 2 mm in length) in contact with FM4–64 dye immediately displayed a PM labeling of cells from the root cap, the rhizoderm (Fig. 2) and the first two cortical layers (Fig. 2b), while labeling of deeper cortical cells was rarely observed (Medicago has 4–6 root cortical layers). This indicates weak dye penetration and/or low access of confocal laser to deeper cell layers. The meristematic zone was not labelled (Additional file 2: Figure S1) possibly be due to its deeper localization or distinct cell wall properties that prevented dye penetration under our conditions [28]. FM4–64 labelling allowed us to visualize four functional zones along the longitudinal axis. Zone 1 corresponds to the root cap and the meristematic active cell division zone (RAM), zone 2 to the root transition zone (RTZ), zone 3 to the elongation zone (EZ), and zone 4 to the growth differentiation zone (GDZ) that harbors root hairs (arrow Fig. 2a). In these zones, PM labeling allowed us to distinguish cells of different sizes and shapes according to their identity and position. Root cap cells, which protect root tip, are elongated and some are released from the cap periphery (arrowheads Fig. 2a). We should note that these root cap cells extend beyond the EZ (zone 3) (Fig. 2a). Both rhizodermal and cortical meristem cells (RAM, zone 1) present isodiametrical shape and division in both directions (Fig. 2b). In zones RTZ to GDZ (zone 2 to 4), cortical cells are larger compared with the rhizodermal ones (Fig. 2b). As expected, the closer the cells are to the root base, the more they are elongated.

In the first minutes, the labeling of the majority of cells presented a well-defined fluorescent contour with exception of root cap cells that contain numerous intracellular fluorescent dots triggering fluorescent signal saturation (Additional file 2: Figure S1 arrow). Because of this complex intracellular labeling we could not take into account the root cap cells for the following study, and endocytosis was thus only monitored in the rhizodermal cells and the first two layers of cortical cells from the RTZ, EZ and GDZ (zones 2, 3 and 4).

**Fig. 3:**
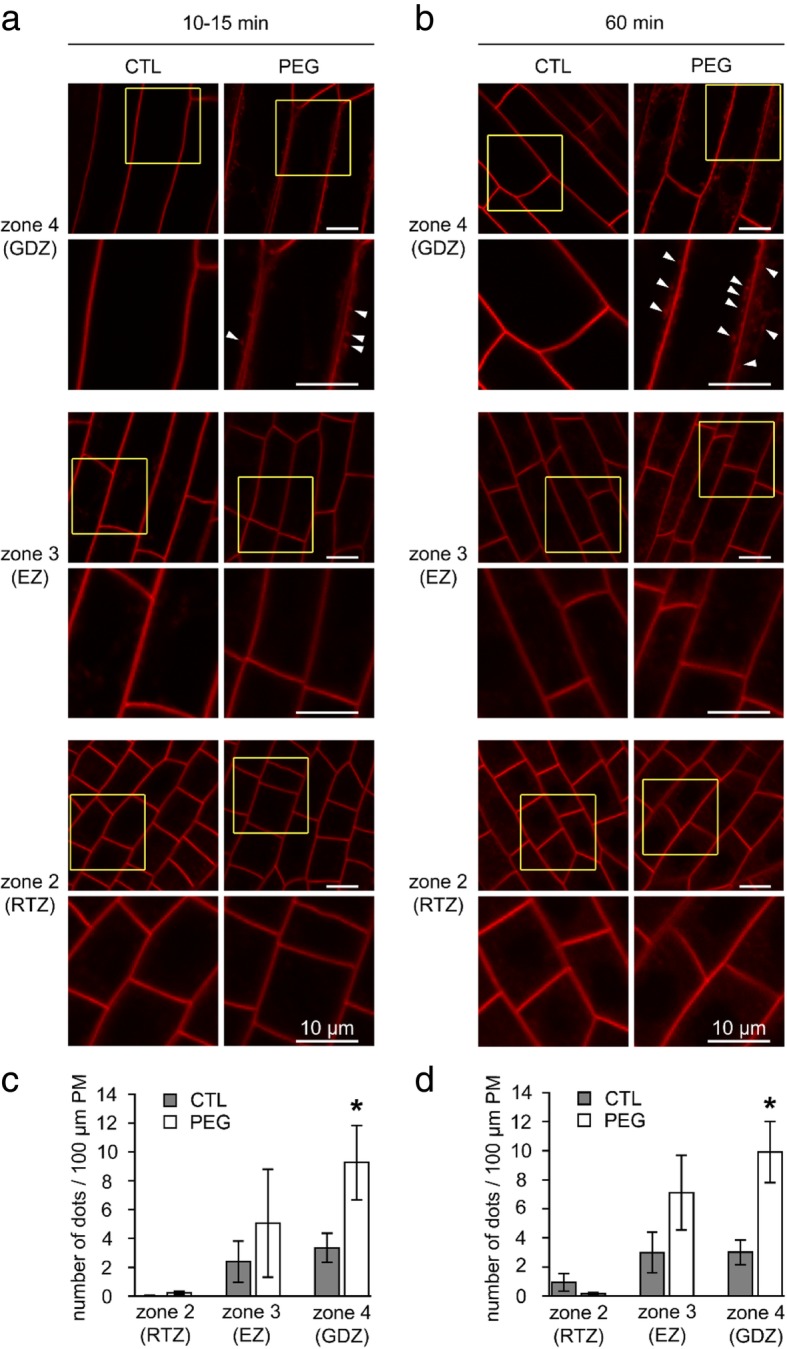
Spatial and temporal stimulation of endocytosis in rhizodermal cells of accession A17 LRs in response to PEG treatment. Representative confocal images of rhizodermal cells of untreated (CTL) or treated roots with 15% PEG (PEG) after (**a**) 10–15 min or (**b**) 60 m in the three root zones (see Fig. 2). For each zone a magnification of inset is shown. Arrowheads indicate endocytic fluorescent dots. **c**, **d** Quantification of the endocytosis expressed in number of fluorescent dots per 100 μm of PM after 10–15 min (**c**) or 60 min (**d**) in control (grey bars) or PEG-treated cells (white bars) according to cell type zone. Values are the means ± SE of 4–6 independent replicates (6–9 roots). Asterisks (*) represent statistical significance of Mann-Whitney test (*p* < 0.05)

In order to reveal how drought is sensed at the cellular level, we first investigated the effect of PEG on root cell endocytosis. Application of 15% PEG 8000 was carried out on well-developed LRs from 7-day germinated A17 seedlings. The presence of FM4–64 labeled fluorescent dots within cells, which reflects endocytosis, was monitored by confocal imaging and quantified using imageJ as previously described in [29]. A rapid and significant increase of FM4–64 internalization was observed in the rhizodermal cells of the upper GDZ zone 4 after only 10–15 min of PEG treatment when compared to untreated roots (Fig. 3a and c) and this internalization was confirmed after 1 hour of treatment (Fig. 3b and d). This stimulation was visible after 1 h in EZ (zone 3), although not significant, but not observed in RTZ (Fig. 3b-c). In cortical cells, endocytosis was not detected after 10 min neither in control nor in PEG-treated cells, but after 1 h, endocytosis was slightly higher in PEG-treated cells even though it occured to a lesser extent than in rhizodermal cells and not significant (Additional file 3: Figure S2).

Taken together, these data show that a greater bulk-flow of endocytosis is induced by PEG in GDZ (zone 4) compared to EZ (zone 3) and RTZ (zone 2), first confirming that endocytic activity depends on cell identity and second suggesting the existence of a longitudinal gradient of stimulated endocytosis from the apex to the elongation zone of rhizodermal cells.

### Mapping of membrane lipid order and packing in rhizodermal cells

To assess the effect of PEG-induced drought on membrane order, we used the di-4-ANEPPDHQ, a membrane fluorescent probe sensitive to local lipid packing, which has been previously used to monitor membrane lipid order in plant cells [30–33]. The spectral properties of the di-4-ANEPPDHQ dye are modified when the membrane environment is changed [34] and a General Polarization (GP) parameter, which defines proportionally the lipid order of the membrane, is calculated from dual-channel images recorded from green and red fluorescence emissions [35].

**Fig. 4:**
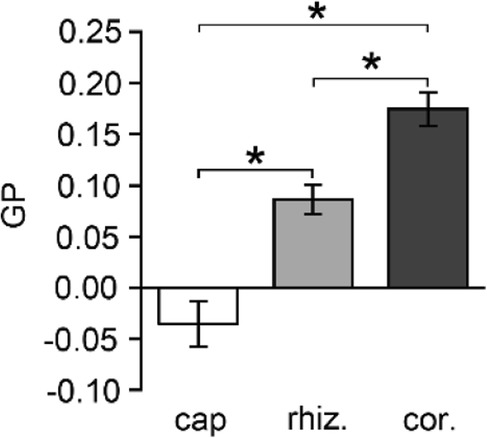
Differential membrane organization in cell types according to their radial distribution. Di-4-ANEPPDHQ GP values were calculated in root cap (cap), rhizodermal (rhiz.) and cortical (cor.) cells of accession A17 in all combined LR zones showing a significant difference along radial delimitation. Values are the mean ± SE of 4 independent replicates (9 roots). Asterisks (*) represent statistical significance of Mann-Whitney test between LR zones (*p* < 0.05)

In *M. truncatula* LRs, the di-4-ANEPPDHQ dye stained well rhizodermal cell membranes and to a lesser extent cortical cells membranes, as seen for FM4–64 labelling. Di-4-ANEPPDHQ fluorescence emissions of both longitudinal and transverse sides of the PM from cap, rhizodermal and cortical cells were thus recorded (Fig. 4). This global analysis -without distinction of longitudinal zones- showed a significant difference in mean GP values between cell types; rhizodermal cells displayed more lipid-ordered domains than root cap cells but less than cortical cells. This suggests that lipid packing varies along the radial axis. The detailed analysis of GP values in the radial root cells after distinction of the longitudinal zones showed the same result (Additional file 4: Figure S3).

**Fig. 5:**
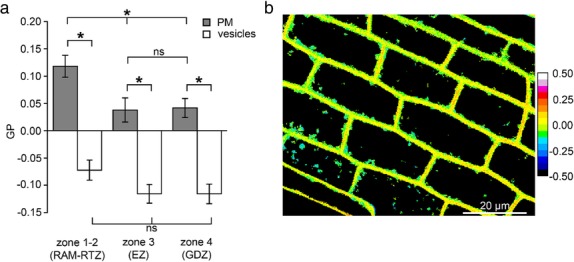
Comparative membrane lipid order between plasma membrane (PM) and intracellular vesicle membrane of A17 untreated rhizodermal cells. **a** GP values of PM (grey bars) and vesicles (white bars) decreased in root zones 3 and 4. GP values of vesicle membranes were lower than the ones in PM. Values are the means ± SE of cells from 3 independent replicates (2 roots per replicate) and 20–30 vesicles per rhizodermal cells. Asterisks (*) represent statistical significance of Mann-Whitney test (*p* < 0.05). ns: nonsignificant. **b** Color LUT (Look Up Table) of a representative image of rhizodermal cells labeled by di-4-ANEPPDHQ from zones 3 and 4. The spectral bar indicates the GP values from the images; purple color represents high GP values and blue color represents low values

Due to the weaker labeling of cortical cells, we focused only on rhizodermal cells thereafter. In order to determine whether lipid packing in the rhizodermal cell membrane depended on the LR zone concerned, GP values were measured for the four distinct longitudinal root zones (Fig. 5a). RAM-RTZ (zone 1–2) cell membranes displayed significant higher GP values than more mature cells from EZ (zone 3) and GDZ (zone 4) that both had similar GP values. In addition, the labeled vesicles within cells possessed similar negative GP values whatever the root zone (Fig. 5a-b). Taken together, these results suggest that in the control condition, intracellular vesicles are composed with more disordered membranes than the PM from which they originate.

### PEG increases lipid order in mature rhizodermal root cells

**Fig. 6:**
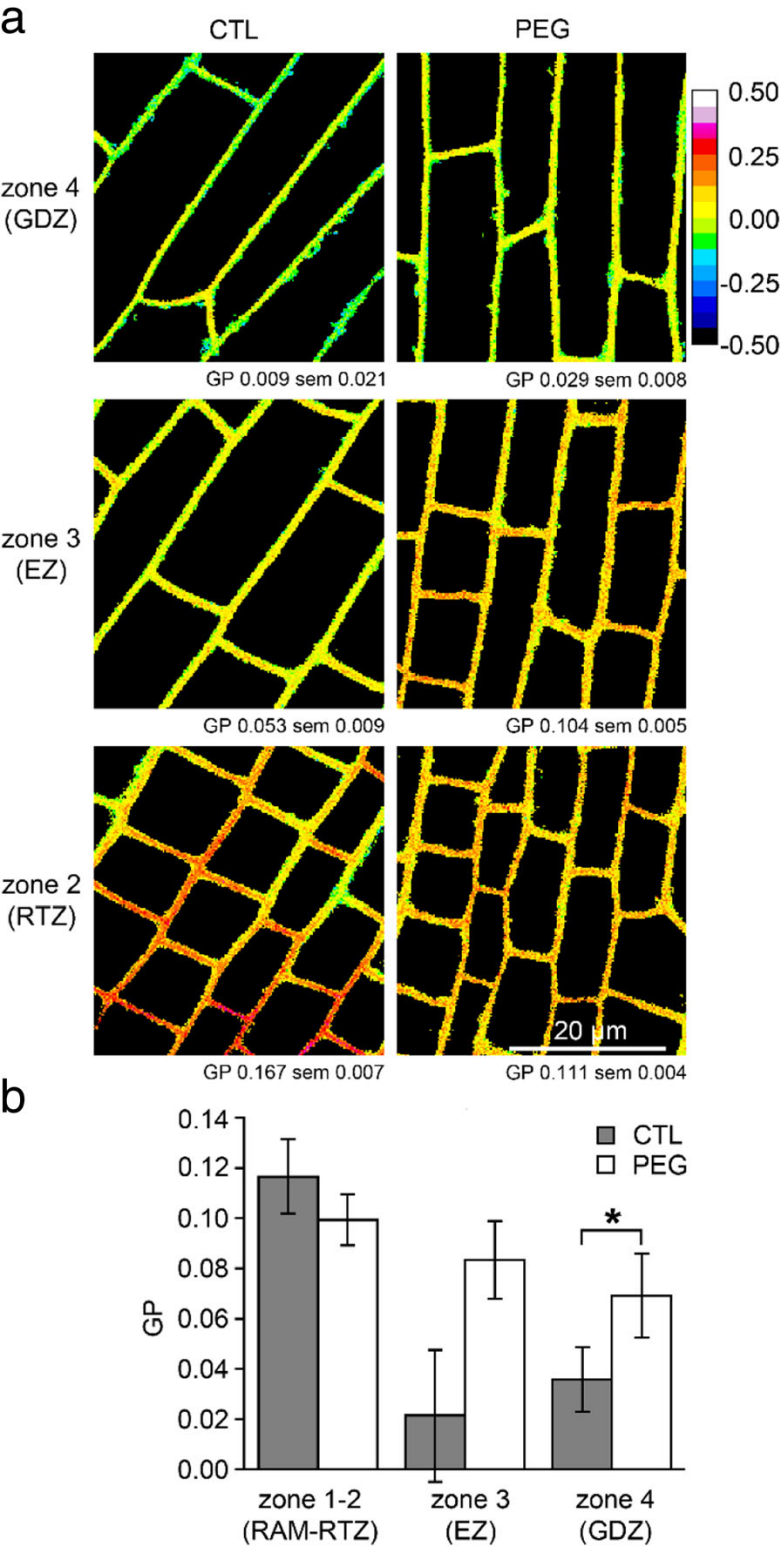
Membrane organization of rhizodermal cells along longitudinal pattern is affected by PEG treatment. **a** Images of rhizodermal cells in three root zone in absence (CTL) or presence (PEG) of 15% PEG. Numbers below images represent the mean of GP value +/− SE in the cells represented in the image. **b** Quantification of GP values calculated in rhizodermal cells in absence (CTL) or presence (PEG) of 15% PEG. Values are the mean ± SE of cells from 9 roots for CTL and 10 roots for PEG treatment. Asterisk (*) represents statistical significance of Mann-Whitney test (*p* < 0.05)

The impact of PEG treatment on the GP parameter was evaluated in rhizodermal cells along the root axis. The distribution of GP intensities are shown with colored-maps of optical sections from representative rhizodermal cells (Fig. 6a). The comparison of the GP mean values between untreated (CTL) and PEG-treated cells (PEG) showed that while RAM-RTZ (zone 1–2) cells are not impacted by PEG, cells from the EZ (zone 3) and GDZ (zone 4) show a PEG-induced increase of lipid ordered domains although this is only significant in GDZ (Fig. 6a-b). As observed for the untreated roots (Fig. 5), intracellular vesicles of PEG-treated cells displayed lower GP values compared with the PM GP (Additional file 5: Figure S4). However, no difference in vesicle GP values was observed between the different root zones (Fig. 5) indicating their differential composition in comparison with the PM.

### Extension of the membrane analyses to a PEG-resistant accession

**Fig. 7:**
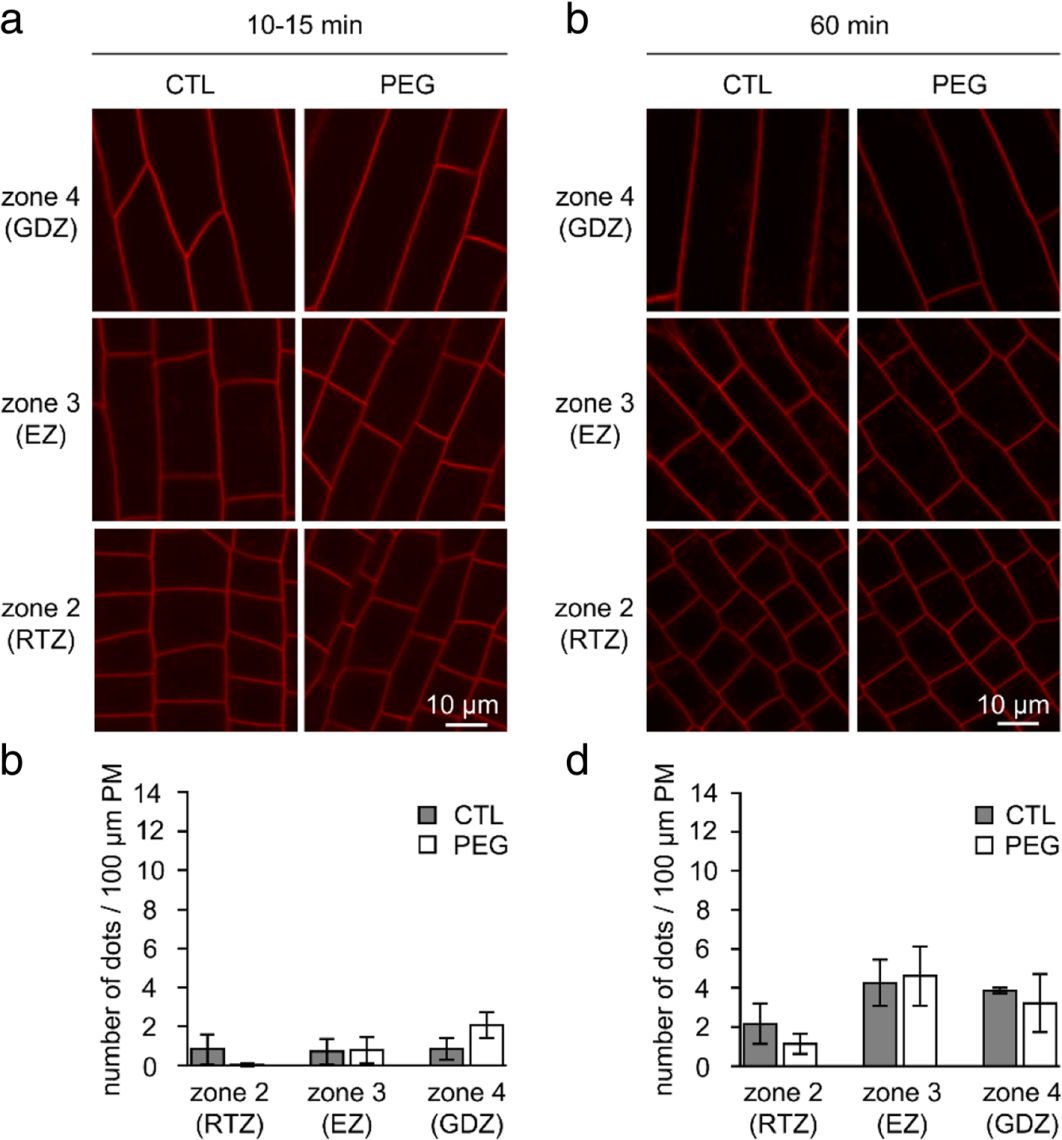
PEG treatment does not induced endocytosis in accession HM298. **a**, **b** Confocal images of rhizodermal cells in the three zones labeled with FM4–64 in absence (CTL) or presence (PEG) of 15% PEG for 10–15 or 60 min. **c**, **d** Quantification of the endocytosis expressed in number of dots per 100 μm of PM after 10–15 min (**c**) or 60 min (**d**) in control (grey bars) or PEG-treated cells (white bars) according to cell type zone. Values are the mean ± SE of cells from 2 to 3 roots

**Fig. 8:**
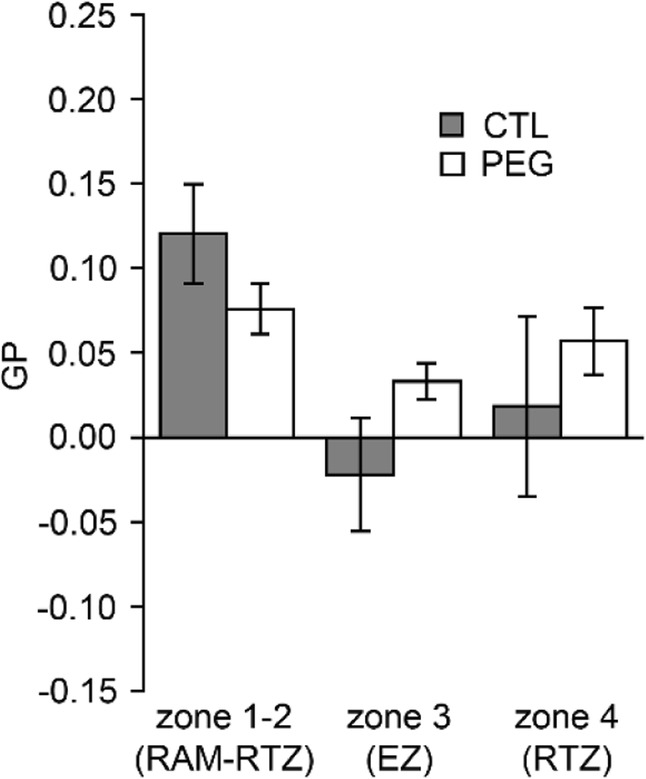
The membrane organization is not modified by 10 min of PEG treatment in accession HM298. Quantification in the three zones of GP values that correspond to the mean ± SE of cells from 4 roots in control (grey bars) or PEG-treated cells (white bars)

We then asked whether the PEG-induced changes in membrane properties are specific to the PEG-sensitive accession. Following the evaluation of the 15 accessions in response to PEG application (Table 1), a resistant accession (HM298), which was also selected as a PEG-resistant accession by [27], was chosen for subsequent analysis of endocytosis and lipid order of plasma membrane and vesicles. Even after 1 h, endocytosis was not affected by PEG treatment in accession HM298 (Fig. 7). Interestingly, this accession did not display a significant PEG-induced modulation of lipid order whatever the root zone (Fig. 8), and in particular in the GDZ, unlike the PEG-susceptible accession A17 used in this study.

## Discussion

The dynamic structure of cellular membranes in eukaryotes is required for their roles in numerous cellular processes. In the present study, we analyzed PM features in LR cells of *M. truncatula* within minutes of exposure to drought stress, as proof of its perception that may reflect the final outcome of plant performance under long-term stress. Indeed, LRs, which are formed post-embryonically, are very responsive to environmental cues [36].

### Endocytosis stimulation under drought stress

Endocytosis, which is an element of intracellular trafficking, is a ubiquitous process in eukaryotic cells that regulates the internalization of PM proteins. This process has not only a pivotal role for plant development and cell function, but also for the control of cell homeostasis in response to environmental cues [37, 38]. This links endocytosis to cell signaling [39, 40].

In the present study, endocytosis was monitored with the FM4–64 dye, which fluoresces only when embedded in a lipid environment such as the PM and endocytic vesicles, as it is integrated on vesicle membranes following endomembrane system-dependent internalization [41–43]. The PM labeling helped us first to distinguish *M. truncatula* A17 cells in terms of identity or functionality, and then allowed us to show that PEG treatment stimulated endocytosis in this accession. Previous studies showed that water (/osmotic) stress induces a reduction in cell surface area, due to the mitigation of turgor pressure, causing internal vesiculation of the PM, together with internalization of extracellular material [44, 45]. In particular, PEG-induced endocytosis of biotinylated extracellular markers has been demonstrated in rice cells [46].

Changes in the osmotic environment of Arabidopsis roots incurred by mannitol, sorbitol or NaCl have been shown to enhance not only the internalization of the FM4–64 dye but also some PM proteins in rhizodermal cells [47, 48]. These hyperosmotic effects have been partially counteracted by genetic and pharmacological interference of clathrin-mediated endocytosis (CME) [48, 49]. This pointed to the involvement of CME as a major mechanism by which PM proteins are internalized in a highly regulated manner [50]. In addition, by disrupting the PM equilibrium, osmotic stress influences the balance between endocytosis and exocytosis acting through the CME [48]. Interestingly, a proteomic study from *Brassica napus* exposed to PEG showed that proteins involved in intracellular trafficking were up-regulated within hours, such as a clathrin heavy chain, the subunit α2 of the clathrin adapter, the syntaxin vam3p, an annexin, a synaptotagmin and some small G rab proteins [51]. These proteins may re-establish the cellular osmotic balance by the compartmentalization of endomembranes or membrane proteins under water stress. A class of membrane proteins involved in water stress are PM aquaporins (PIPs), which enable the flux of water through membranes. Notably, a genome-wide association analysis of PEG-related and biomass phenotypic variation in *M. truncatula* has identified the aquaporin PIP2 as the second most significantly associated gene with shoot biomass [27]. Interestingly, after hyperosmotic salt stress, an internalization of PIPs has been demonstrated in Arabidopsis rhizodermal and cortical root cells [47]. This internalization may contribute to protect cells against dehydration by reducing aquaporin abundance at the PM. The PIPs are not only internalized by CME [52], but a clathrin-independent endocytosis (CIE) of PIPs may also occur [53]. Indeed, the less-studied CIE pathway seems to be dependent on sterol- and sphingolipid-enriched membrane microdomains leading to more ordered domains. By using probes reflecting CME and CIE pathways, a stratified distribution of these processes in Arabidopsis roots under salt stress was observed [54]. Indeed, CME that internalizes transmembrane proteins was found in all cell layers, while CIE that mobilized lipids and lipid-anchored proteins, but not transmembrane protein, was restricted to the rhizodermal layer. By contrast, in saline stress, a third pathway, that is a non-discriminatory CIE in its choice of cargo (lipids or proteins), operated in all layers of the root. This can be considered as a reshuffle of the nature of the endocytic pathways due to the influence of an environmental stimulus on the physical state of the membrane.

### Physical state of membranes of “resting” root cells

The mapping of lipid order of membranes in rhizodermal cells of *M. truncatula* A17 highlighted different membrane properties in different root zones. These lipid order-rich domains are thought to intervene in temporal and spatial organization of cell-specific processes. This differential lipid order was also observed in cell types of Arabidopsis root labeled with di-4-ANEPPDHQ dye [33]. We showed that in Medicago, as in Arabidopsis [33], cortical cells displayed higher lipid order than rhizodermal cells, and that cells from the distal root zones (RAM and RTZ; zones 1 and 2) had higher ordered membranes than the elongated and differentiated cells from the GDZ (zone 4). The physical state of the membrane relies on its composition, thus modulation of membrane lipid order may be explained in part by differential lipidome distribution between meristematic and mature cells as observed in soybean [55] and barley roots [56]. Some of these lipids are involved with compartmentalization of intrinsic proteins within lipid microdomains, and are critical in creating platforms for a necessary signal transduction during division and growth (proposed in [17, 33]).

Moreover, in the present study, GP values of PM of rhizodermal cells were statistically higher than those of membranes from intracellular vesicles. This differential order degree between membrane surface and the derived vesicles was also seen in Arabidopsis roots [32] indicating a modification of membrane composition as expected.

### Drought stress modifies physical state of root cell membranes

Medicago A17 rhizodermal cells showed an increased PM lipid order in response to PEG, reflecting changes in the PM physical state. It is well-known that during hyperosmotic conditions, bacterial osmosensors at the cell surface are activated by changes in physical state / protein-protein interactions [57, 58]. Similarly, in the plant *Pyrus betuloefolia*, the lipid microviscosity of the PM increased in response to PEG while the PM fluidity decreased, indicating an influence of water stress on the physical state of the membrane [59]. In a plant growth-promoting bacterium (*Azospirillum brasilense*), PEG decreased the fluidity a few minutes after treatment, influencing the perception of the water stress by the bacteria and after 1 h, this effect was offset by an adjustment in lipid composition, contributing to the homeostasis of membrane fluidity under water deficit [60]. Similarly, a recent study showed that a NaCl-mediated hyperosmotic stress modified the spatial distribution and abundance of lipid species in roots of barley seedlings [56]. Interestingly, these stress-induced changes in lipid abundance were more important in the maturation zone than other root zones, suggesting the existence of a stress gradient from the root cap to the maturation zone [56].

In accordance with the above literature, we observed an increase in lipid-ordered state in membranes of Medicago rhizodermal cells from GDZ (zone 4) while PEG did not significantly influence the other zones. Thus, the GDZ of rhizodermal cells, which is the start of the maturation zone, may represent a PEG-responsive zone associated to perception because in the present study the extent of water stress stimulated both endocytosis rate and lipid order. This zone contains differentiated cells with root hairs that are extensions of rhizodermal cells and greatly increase the absorptive surface for collecting water. In the present study, we did not analyze the lipid order of root hairs as they were sometimes damaged by recovery from the pouch paper wick, but such a study has been performed in Arabidopsis [33] showing polar hair growth was associated with a polar distribution of highly ordered PM.

We already suggested that PM compartmentalization may modify biochemical functions, in particular those responsible for intracellular signaling. This could allow delivery and retrieval of membrane molecules, that is, exocytosis and endocytosis, at specific membrane locations to be influenced by or influence back the formation of membrane domains. Surprisingly, the lipid order of the intracellular vesicle membranes was not modified in response to PEG, indicating the stability of endocytic compartment composition. In addition, the study of lipid order in Arabidopsis root cells (without stress) identified the vesicles as mostly clathrin-coated [32]. Accordingly, to go further in our study, an analysis of the distinctiveness of endocytosis could be performed in untreated and PEG-induced Medicago by the use of markers of the endocytotic pathways such as the subunit σ2 of the clathrin adaptor [61] or application of dedicated endocytic inhibitors such as tyrphostin A23 [42] or ikarugamycin [62] that blocks clathrin-coated pits on the PM and vesicle formation.

### Does a link exist between ecophysiological traits of drought tolerance and membrane dynamics?

The use of 15 accessions identified as highly genetically diversified [26] enabled us to observe contrasted responses to PEG treatment in terms of plant biomass allocation strategy towards shoots and roots, and in terms of root architecture plasticity. In this study no direct link could be established between root architecture plasticity of several traits and drought resistance (evaluated as a loss of total biomass), although some root traits such as specific root length, number of LRs or insertion angles of LRs are known to play a role in water uptake under water stress [7]. This reflects the necessity of combining architectural and morphological root traits with cellular processes in order to decipher key mechanisms enhancing drought resistance.

In the present study, accession A17 was qualified as drought susceptible due to the significant PEG-induced reduction of both root and shoot biomasses, while HM298 was considered as drought-tolerant with the maintenance of plant growth under PEG conditions. In A17, rhizodermal cells presented a short-time stimulation of endocytosis after PEG treatment while even after 1 h this was not the case for HM298. Such a differential uptake between genotypes was observed in rice cells using biotinylated markers in reponse to NaCl stress, while tolerance to sorbitol was correlated with an increased uptake [46]. It is tempting to speculate that PM water channels are more regulated by their internalization in A17 than in HM298, limiting the intensive cell dehydration, as was observed in Arabidopsis [63]. This is also in accordance with the modulation of root architecture observed in HM298 (and not in A17), that could enhance water acquisition to compensate water loss by PM aquaporin. Indeed, the number of LRs was increased under PEG conditions in this accession and could thus explain its drought tolerance as reviewed in [64].

We also showed that A17 cells displayed a higher increase of the lipid order of membrane of rhizodermal cells, confirming a link between the two processes - endocytosis and lipid order - for stress perception in a “susceptible” genotype. Further investigations are needed to understand the relationship between PEG-induced membrane dynamics and the degree of drought tolerance, by extending the analysis to the other *M. truncatula* accessions representing the whole range of tolerance.

Indeed, we may hypothesize that these early membrane responses, namely endocytosis or lipid packing, may facilitate stress perception or, in reverse, the perception (by another way) may trigger membrane dynamics by mechanosensing. These two membrane processes - linked or not - may be seen as the first steps in the plant’s response to drought stress and can be extended to other abiotic stresses similarly to what was proposed for biotic stress [31, 42]. Finally, our results pave the way to go further with the *M. truncatula* accessions that offer a high level of genetic diversity and high-density molecular marker maps to better understand the behavior of plant under water stress.

## Conclusions

This study indicates that cell membranes are not simply passive barriers liable to be damaged during environmental cues, but are involved in cellular responses via the modulation of intracellular signaling. How membrane dynamics of root cells perceive drought and transmit signals to upstream ecophysiological responses via transduction mechanisms is still an open question.

By using confocal imaging of fluorescent dyes, we provide evidence for differential behavior (/properties) of root cell membranes according to their age and location within the organ in a “susceptible” genotype. By analyzing the behavior of cell PM under PEG-induced drought stress we have highlighted a major modification of the cells in the GDZ mature zone.

## Methods

### Plant material and growth

Seeds from the *Medicago truncatula* CC16 core collection [26], Jemalong A17, HM026 and HM298 were kindly provided by the *M. truncatula* Genetic Resources Centre (Montpellier, France, https://www1.montpellier.inra.fr/BRC-MTR/). Seeds were scarified for 10 min in 99% sulfuric acid (H_2_SO4), rinsed four times with sterile water, sterilized in 0.6% sodium hypochloride for 20 min and rinsed four times with sterile water. Seeds were then germinated on plate containing a cellulose sheet soaked with sterile water for 3 days in the dark. Over the 16 genotypes from the CC16 Medicago core collection, only 12 successfully germinated and were subsequently studied (Additional file 1: Table S1). Germinated seedlings were then transferred to autoclaved pouches (https://mega-international.com/tech-info/) previously soaked with 8 mL of nutritive solution [1.87 mM KNO_3_, 0.57 mM K_2_HPO_4_, 2.82 mM Ca(NO_3_)_2_, 1 mM MgSO_4_, 2.5 mM NaNO_3_, 0.05 mM EDTA-FeNa and oligoelements] as described in [65]. Three seedlings per pouch (Fig. 1) and 10 replicate pouches per accession were used. The lower part of each pouch was wrapped in an aluminum cover in order to keep roots in darkness, and pouches were then placed in upright position in a growth chamber under a 16 h photoperiod (Photosynthetically Active Radiation maintained at 197 μE/m^2^/s with fluorescent light) and a temperature of 22/20 °C (day/night).

### PEG treatment

For root system architecture evaluation of the 15 accessions, plants were grown in pouches during 12 days. After 4 days in pouches (corresponding to 7 days after germination), half of the pouches received 3 mL of nutrient solution while the other half received 3 mL of nutrient solution supplemented with 15% PEG-8000. The expected water potential of the nutrient solution supplemented (− 0.3 MPa) or not (− 0.007 MPa) with PEG according to [66] was checked in C52 sample chambers coupled to a Wescor HR-33 T Dew Point Microvoltmeter (Wescor Inc., Logan, UT, USA) at 20 °C. Pouches were checked daily and 2 mL of either nutrient solution or nutrient solution supplemented with PEG were added to the pouches in order to compensate the evapotranspiration of the seedlings in untreated (CTL) or PEG-treated pouches, respectively.

For microscopical analysis, after 4 days in pouches, LRs were collected and transferred in Eppendorf tubes containing the nutrient solution. For PEG treatment, LRs were placed in nutrient solution containing 15% PEG-8000 (PEG-8000/nutritive solution, w/v) for either 10–15 min or 1 h. LRs that were not subjected to PEG were used as control (CTL).

### Ecophysiological parameters analysis

For each *Medicago truncatula* accession, 10 to 15 individual seedlings were analysed after 12 days growth in pouches. Root system was scanned (400 dpi) and image analysis was performed using both Smartroot toolbox under ImageJ software [67] and WinRhizo software version 2012b (Regent Instruments Inc., Canada) by separing the main root from LRs (Fig. 1a). Total projected root length, number of LRs and average insertion angles of LRs were thus evaluated. Then seedings were harvested and roots were separated from shoots. Biomasses of shoot and root were measured after oven drying sample at 80 °C for 48 h. Specific root length was then calculated as the ratio between total root length and root biomass.

### Staining with FM4–64 and endocytosis analysis

Plasma membrane staining and internalization were evaluated with the styryl dye FM4–64 (Invitrogen life Technologies #T-13320) that embeds into membrane. The stock solution of FM4–64 was prepared at 2.125 mM in water and stored at − 20 °C. LRs were treated with 4.25 μM FM4–64 added in nutritive solution in presence or not of 15% PEG, and incubated at 25 °C for 10–15 min or 1 h. Images of root cells from different functional zones were captured with a SP2 Leica confocal laser scanning microscope using a 40x oil immersion objective (NA = 1.25). FM4–64 was excited using a 488 nm laser and the fluorescence collected in the range of 600–700 nm. Quantification of FM4–64 internalisation was carried out as described in [29]. Briefly, intracellular fluorescent dots were scored and scaled to 100 μm of PM using Image J software (http://rsbweb.nih.gov/ij/). At least 50 cells were analyzed per LR (*n* = 2 to 9).

### Di-4-ANEPPDHQ staining and GP processing

The ratiometric dye di-4-ANEPPDHQ (Invitrogen life Technologies #D36802) allows the evaluation of membrane organization as it differentiates coexisting liquid-ordered phases from liquid-disordered phases. The stock solution of di-4-ANEPPDHQ was prepared at 1.5 mM in dimethylsulfoxide (DMSO) and stored at − 20 °C. For staining, LRs were excised from 7-day old seedlings and placed in medium [nutritive solution +/− 15% PEG] containing 3 μM di-4-ANEPPDHQ dye for 5 min. LRs were washed for 1 min in medium without the dye and transferred onto glass slide with 80 μL of medium. A 40x oil immersion objective (NA = 1.25) of a Leica SP2 confocal laser scanning microscope was used for the imaging of 4-di-ANEPPDHQ-labeled cells. The LRs were excited using a 488-nm laser and the emission range of two channels was set to 540–560 nm (green fluorescence) and 650–670 nm (red fluorescence) reflecting higher and lower membrane lipid order, respectively. Identical microscope settings were maintained for quantitative imaging of membranes. For each cell, longitudinal and transverse sides of the PM and/or membrane vesicles were analyzed. Recorded images were treated with Image J using the Owen macro [35] to generate the GP (General Polarization) parameter proportional to membrane order as followed: GP = (I_540–560_ – I_650–670_) / (I_540–560_ + I_650–670_), where, I_540–560_ and I_650–670_ corresponds to fluorescence intensities collected by the two microscopic channels.

### Statistical analyses

For ecophysiological traits, measured on 10–15 seedlings, analysis of variance were performed (type III sum of squares) and means were compared using a Tukey’s HSD (honest significant differences) test at the 0.05 probability level, using R software (https://www.r-project.org/). For endocytosis or lipid order analysis, significance between samples were evaluated using the Mann-Whitney U-test in PAST software (https://folk.uio.no/ohammer/past/).

## Additional files

Additional file 1: **Table S1.** Values of the traits of *Medicago truncatula* accessions after 12 days in pouches without PEG (none) or with PEG treatment on biomasses and root architectural traits. Mean ± standard deviation of 15 seedlings. (XLSX 20 kb)

Additional file 2: **Figure S1.** Confocal image in the deeper zones of a LR of accession A17. Meristematic zone (m), endodermis, and vessel (v) are not reached by the FM4–64 dye. Intense and saturated FM4–64 intracellular labeling is found in root cap cells (arrow). (TIF 1067 kb)

Additional file 3: **Figure S2.** Spatial and temporal stimulation of endocytosis in cortical cells of accession A17 LRs in response to PEG treatment. (**a**, **b**) Confocal images of rhizodermal cells of untreated (CTL) or treated roots with 15% PEG (PEG) after (**a**) 10–15 min or (**b**) 60 min the three root zones (see Fig. 2). (**c, d)** Values are the mean ± SE of cells from 7 roots after (**c**) 10–15 min or (**d**) 60 min in control (grey bars) or PEG-treated cells (white bars) according to cell type zone. (TIF 1860 kb)

Additional file 4: **Figure S3.** Differential membrane organization in cell types according to their radial and longitudinal distribution. Di-4-ANEPPDHQ GP values were calculated in root cap (cap), rhizodermal (rhiz.) and cortical (cor.) cells of accession A17 in the four LR zones. Values are the mean ± SE of 4 independent replicates (9 roots). Asterisks (*) represent statistical significance of Mann-Whitney test (*p* < 0.05) between each cell type in a same zone. ns: non significant. (TIF 126 kb)

Additional file 5: **Figure S4.** Comparative membrane organization between plasma membrane (PM) and vesicle membrane in rhizodermal cells of accession A17 treated by 15% PEG. GP values of vesicle membranes (white bars) were lower than the ones in PM (grey bars). Values are the means ± SE of 3 independent replicates (2 roots per replicate) and 20–30 vesicles per rhizodermal cells. Asterisks (*) represent statistical significance of Mann-Whitney test (*p* < 0.05). ns: non significant. (TIF 112 kb)

## Abbreviations

CIE: Clathrin-Independent Endocytosis
CME: Clathrin-Mediated Endocytosis
GP: General Polarization
LR: Lateral Root
PEG: Polyethylene Glycol
PM: Plasma Membrane
