# Supplementary figures and images for: Drought stress stimulates endocytosis and modifies membrane lipid order of rhizodermal cells of Medicago truncatula in a genotype-dependent manner
Source: BMC Plant Biol. 2019 May 28;19:221. doi: 10.1186/s12870-019-1814-y (PMC6537417; doi:10.1186/s12870-019-1814-y)

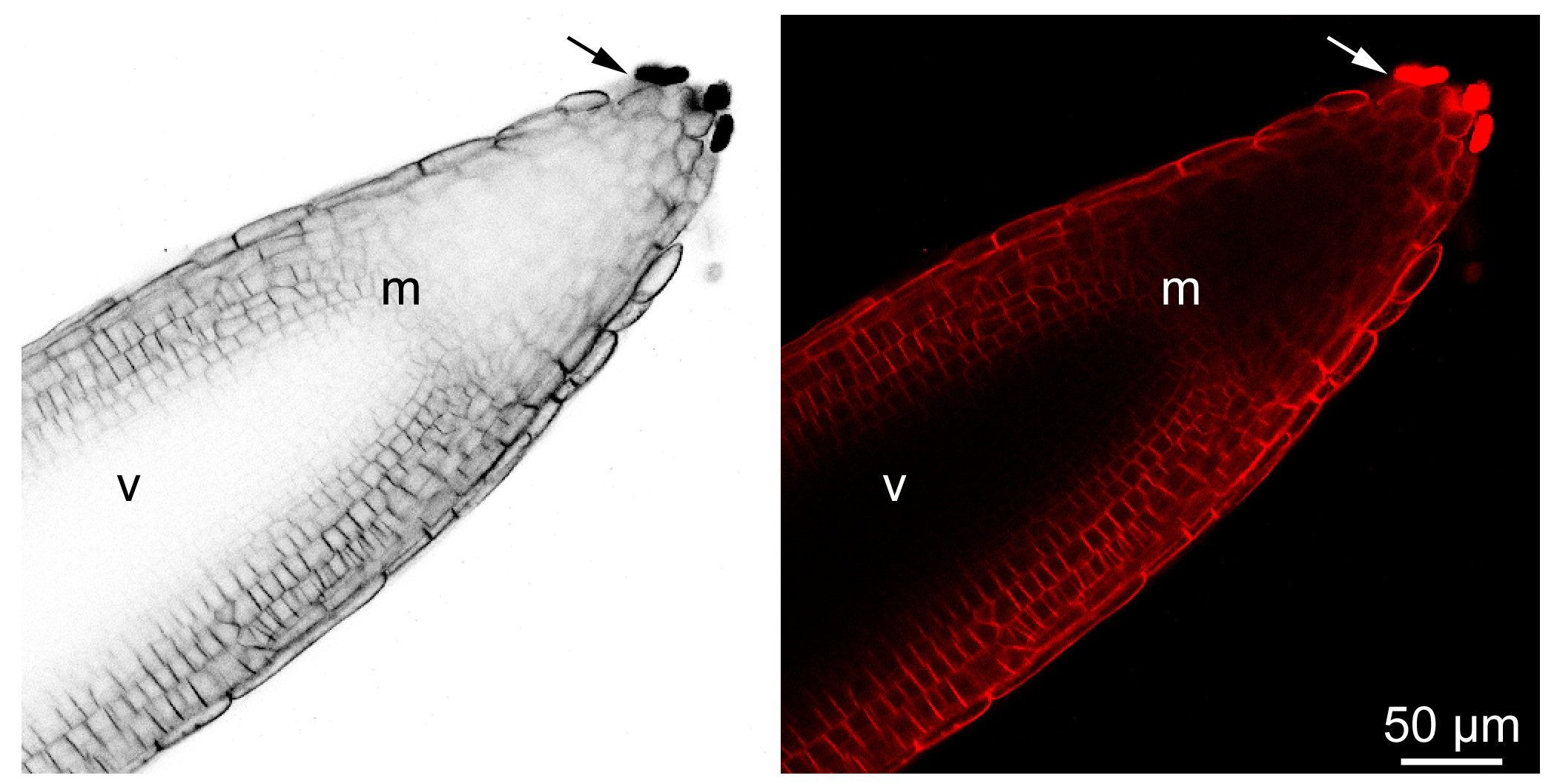

Supplement: Supplementary file 2 — Figure S1. Confocal image in the deeper zones of a LR of accession A17. Meristematic zone (m), endodermis, and vessel (v) are not reached by the FM4–64 dye. Intense and saturated FM4–64 intracellular labeling is found in root cap cells (arrow). (TIF 1067 kb) [file 12870_2019_1814_MOESM2_ESM.tif]

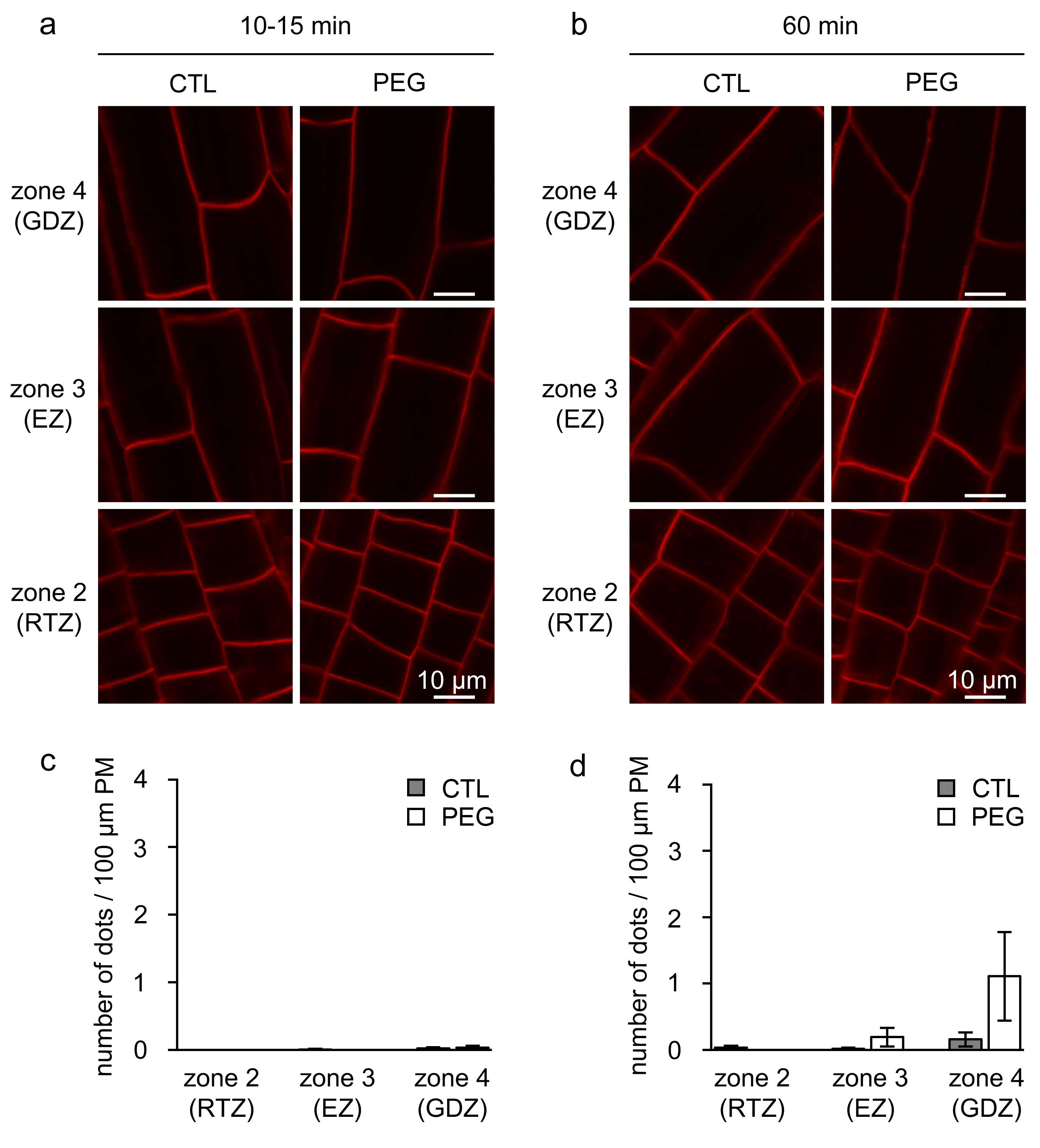

Supplement: Supplementary file 3 — Figure S2. Spatial and temporal stimulation of endocytosis in cortical cells of accession A17 LRs in response to PEG treatment. (a, b) Confocal images of rhizodermal cells of untreated (CTL) or treated roots with 15% PEG (PEG) after (a) 10–15 min or (b) 60 min the three root zones (see Fig. 2). (c, d) Values are the mean ± SE of cells from 7 roots after (c) 10–15 min or (d) 60 min in control (grey bars) or PEG-treated cells (white bars) according to cell type zone. (TIF 1860 kb) [file 12870_2019_1814_MOESM3_ESM.tif]

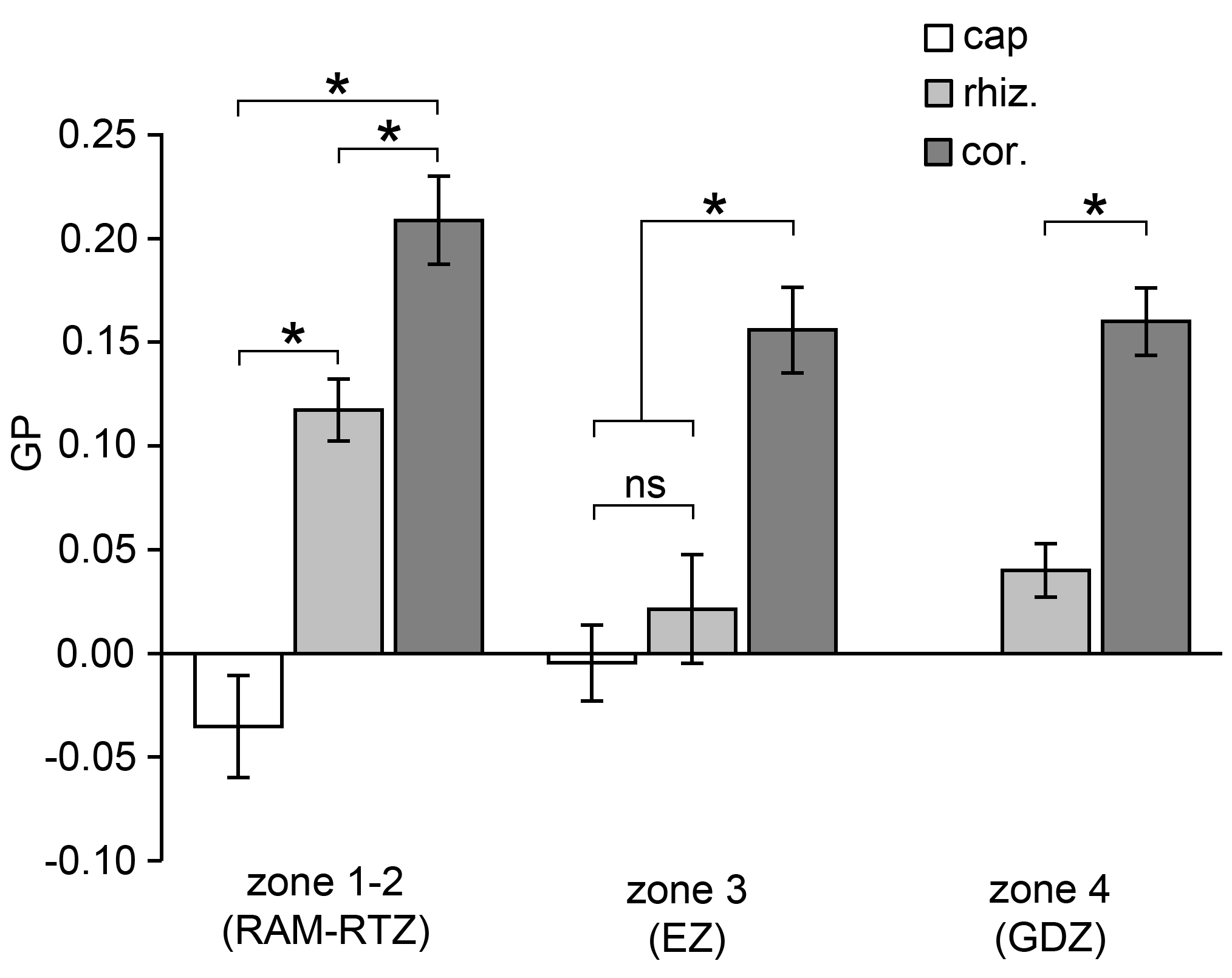

Supplement: Supplementary file 4 — Figure S3. Differential membrane organization in cell types according to their radial and longitudinal distribution. Di-4-ANEPPDHQ GP values were calculated in root cap (cap), rhizodermal (rhiz.) and cortical (cor.) cells of accession A17 in the four LR zones. Values are the mean ± SE of 4 independent replicates (9 roots). Asterisks (*) represent statistical significance of Mann-Whitney test (p < 0.05) between each cell type in a same zone. ns: non significant. (TIF 126 kb) [file 12870_2019_1814_MOESM4_ESM.tif]

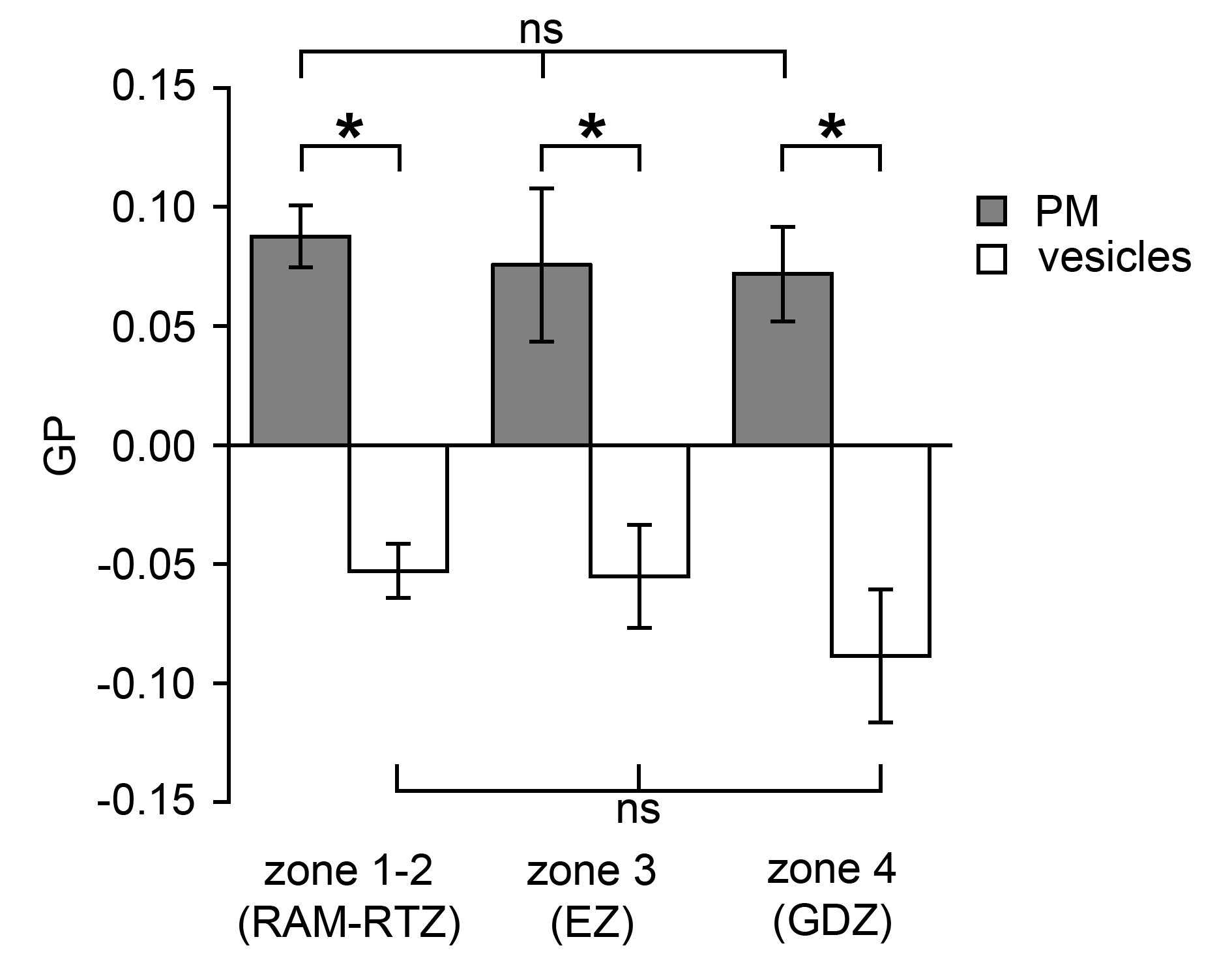

Supplement: Supplementary file 5 — Figure S4. Comparative membrane organization between plasma membrane (PM) and vesicle membrane in rhizodermal cells of accession A17 treated by 15% PEG. GP values of vesicle membranes (white bars) were lower than the ones in PM (grey bars). Values are the means ± SE of 3 independent replicates (2 roots per replicate) and 20–30 vesicles per rhizodermal cells. Asterisks (*) represent statistical significance of Mann-Whitney test (p < 0.05). ns: non significant. (TIF 112 kb) [file 12870_2019_1814_MOESM5_ESM.tif]
